# Clinical and biological characteristics of cervical neoplasias with FGFR3 mutation

**DOI:** 10.1186/1476-4598-4-15

**Published:** 2005-05-03

**Authors:** Christophe Rosty, Marie-Hélène Aubriot, David Cappellen, Jérôme Bourdin, Isabelle Cartier, Jean Paul Thiery, Xavier Sastre-Garau, François Radvanyi

**Affiliations:** 1 Département de Pathologie, Institut Curie, Section Médicale, 26 rue d'Ulm, 75248 Paris Cedex 05, France; 2 UMR 144, CNRS – Institut Curie, Section de Recherche, 26 rue d'Ulm, 75248 Paris Cedex 05, France; 3 Laboratoire du Dr René Cartier, 20 rue des Cordelières, 75013 Paris, France

## Abstract

**Background:**

We have previously reported activating mutations of the gene coding for the fibroblast growth factor receptor 3 (FGFR3) in invasive cervical carcinoma. To further analyze the role of FGFR3 in cervical tumor progression, we extended our study to screen a total of 75 invasive tumors and 80 cervical intraepithelial neoplasias (40 low-grade and 40 high-grade lesions).

**Results:**

Using single strand conformation polymorphism (SSCP) followed by DNA sequencing, we found FGFR3 mutation (S249C in all cases) in 5% of invasive cervical carcinomas and no mutation in intraepithelial lesions. These results suggest that, unlike in bladder carcinoma, FGFR3 mutation does not or rarely occur in non invasive lesions. Compared to patients with wildtype FGFR3 tumor, patients with S249C FGFR3 mutated tumors were older (mean age 64 *vs*. 49.4 years, *P *= 0.02), and were more likely to be associated with a non-16/18 HPV type in their tumor. Gene expression analysis demonstrated that FGFR3 mutated tumors were associated with higher FGFR3b mRNA expression levels compared to wildtype FGFR3 tumors. Supervised analysis of Affymetrix expression data identified a significant number of genes specifically differentially expressed in tumors with respect to FGFR3 mutation status.

**Conclusion:**

This study suggest that tumors with FGFR3 mutation appear to have distinctive clinical and biological characteristics that may help in defining a population of patients for FGFR3 mutation screening.

## Background

Cervical cancer is the second leading cancer in women worldwide and a common cause of death among women in developing countries where 80% of cases occur [1]. Invasive cervical carcinoma develops through a well-defined progression model. Cervical intraepithelial neoplasia (CIN) is the premalignant lesion that always precedes invasive squamous cell carcinoma [2]. These precursor lesions are subdivided into 3 grades (CIN I-III) or 2 grades (low-grade squamous intraepithelial lesions, LSIL and high-grade squamous intra-epithelial lesions, HSIL). Half of low-grade lesions spontaneously regress within 6 months although 10–20% of high-grade lesions may progress to invasive carcinomas [3]. Molecular epidemiologic studies clearly demonstrated that sexually transmitted infection by HPV (human papillomavirus) is the principal cause of cervical carcinoma. Fifteen HPV types are considered as high-risk HPV and are associated with a higher risk of developing invasive cervical carcinoma from squamous intraepithelial lesions [4]. However, HPV infection is not sufficient to transform the normal cervix epithelial cells to invasive carcinomas and several additional events are necessary. Only a few genetic alterations have been reported in cervical carcinoma so far.

We previously reported specific FGFR3 missense mutations in 3 out of 12 invasive cervical carcinomas [5]. FGFR3 belongs to a family of structurally related tyrosine kinase receptors encoded by four different genes (*FGFR1-4*). FGFRs are glycoproteins composed of two or three extracellular immunoglobulin (Ig)-like domains, an hydrophobic transmembrane region and a cytoplasmic part that contains the tyrosine catalytic site. FGFRs are present as inactive monomers on the cell surface, upon ligand binding FGFRs dimerize, autophosphorylate and are able to transmit a series of intracellular signals [6]. An alternative splicing event in the second half of the juxtamembrane Ig-like domain of FGFR3 generates two mutually exclusive isoforms : FGFR3b the main form expressed in epithelial cells and FGFR3c the main form expressed in chondrocytes. Germinal activating FGFR3 mutations result in craniosynostoses and dwarfing chondrodysplasias of varying severity (hypochondroplasia, achondroplasia, SADDAN and thanatophoric dysplasia). Strikingly the same activating mutations have been reported at the somatic level in several types of cancer: multiple myeloma, bladder and cervical carcinomas. Very frequent in bladder carcinoma, particularly in non invasive papillary tumours (pTa tumors) (70% of cases harbor mutations), FGFR3 mutations are more rare in multiple myeloma and cervical carcinomas [5,7,8].

The goal of this work is to extend our previous study to screen a total of 75 patients for FGFR3 mutations and to identify clinical and/or pathological features associated with FGFR3 mutation. We also asked whether FGFR3 mutation could occur at earlier stages of cervical tumor progression, like in bladder tumors for which the highest rate of mutation is for low-stage non invasive pTa tumors. We thus analyzed 80 squamous intra-epithelial lesions (40 LSILs and 40 HSILs).

## Results

### FGFR3 mutation in invasive cervical carcinoma and squamous intraepithelial lesions

To extend our previous report of FGFR3 mutation in 3 of 12 cervical carcinomas [5], we selected 63 additional cases for a total of 75 screened DNAs. Those patients had the same characteristics than the initial cohort of 12 patients. SSCP analysis was performed on exons 7, 10, 15, and 20 of FGFR3, followed by direct DNA sequencing for cases with abnormal SSCP profiles. We found one additional case with FGFR3 mutation. Taken together, our study showed FGFR3 mutation in 4 of 75 invasive cervical cancer (5%) (Table 1). All mutations were C to G transversion at nucleotide 746, which changed serine 249 to cysteine. This S249C mutation creates a cysteine residue in the extracellular domain of the FGFR3 receptor.

**Table 1:** FGFR3 mutations in 75 invasive cervical carcinoma samples

**Sample**	**Age**	**Stage**	**Histology**	**HPV**	**Codon**	**Mutation**	**Predicted effect**
6.96.1	65	IB	SCC	+ *	249	TCC → TGC	Ser → Cys
4.139	69	IIB	SCC	33	249	TCC → TGC	Ser → Cys
4.13	64	IIB	SCC	16	249	TCC → TGC	Ser → Cys
7.79.1	58	IIB	SCC	+ *	249	TCC → TGC	Ser → Cys

* Tumors with HPV DNA sequences of undetermined type (other than HPV 6, 11, 16, 18, 31, 33, 35, 39, 42, 45, 52 and 58). SCC : Squamous Cell Carcinoma

To further analyze the role of FGFR3 in cervical tumor progression, we investigated whether FGFR3 mutations are restricted to invasive carcinoma or may occur in squamous intraepithelial lesions. Hence we analyzed DNA from 40 LSILs and 40 HSILs. SSCP revealed no FGFR3 mutation in any of these cases. For all SSCP experiments, positive controls carrying all somatic FGFR3 mutations previously identified in tumors have been included. The rate of FGFR3 mutation in invasive cervical carcinoma is significantly different from the one in squamous intraepithelial lesions (P = 0.036, chi-square test).

### Overall FGFR3 mutation rate in invasive cervical carcinoma

Studies about FGFR3 mutation in cervical cancer reported various rates of mutation, ranging from 0% to 25%. In order to evaluate an overall mutation rate, we added all results. Hence, we searched for all published papers on FGFR3 mutations in cervical cancer, by interrogating Medline with the terms "cervical cancer" and "FGFR3" (Table 2). Including this work, 6 studies analyzed a total of 349 cervical cancer samples and found 6 FGFR3 mutations (1.7%) [5,9-12]. All mutations were S249C missense mutations.

**Table 2:** Total number of cervical carcinoma cases screened for FGFR3 mutation, as of august 2004.

**Study**	**Method**	**Total number of cases**	**Mutated cases (percentage)**
Cappellen et al. [5]	SSCP (entire coding region)	12	3 (25%)
Yee et al. [9]	Direct sequencing (exon 7)	104	0
Wu et al. [10]	Direct sequencing (exons 7, 10, 13, 15, and 19)	51	1 (2%)
Dai et al. [11]	Amplification created restriction site methodology for S249C mutation	91	0
Sibley et al. [12]	Direct sequencing (exons 7, 10, and 15)	28	1 (3.5%)
Present study	SSCP (exons 7, 10, and 15)	63	1 (1.5%)

Total		349	6 (1.7%)

### Patients with FGFR3 mutation are older that patients with wild-type FGFR3

Clinical and pathological features were retrieved from patient's charts and correlated with FGFR3 status (Table 1). The 4 patients with S249C FGFR3 mutation tumor were significantly older at time of diagnosis than patients without the S249C FGFR3 mutation in the tumor (mean age 64 vs. 49.4 years, *P *= 0.02, Mann-Whitney U test). Clinical stage was IIB for 3 patients and IB for 1 patient. One patient died from the disease after 19 months. Follow-up information for the other 3 patients was available only for 3 months, 47 months, and 81 months, with no sign of relapse.

All 4 mutated tumors were well-differentiated invasive squamous cell carcinomas. HPV DNA of various types was detected in all cases : one HPV16, one HPV33 and two HPV of undetermined type (other than HPV 6, 11, 16, 18, 31, 33, 35, 39, 42, 45, 52 and 58). Distribution of HPV types associated with FGFR3 mutated tumors was rather different than the expected distribution in squamous cell cervical carcinoma. The prevalence of HPV16, HPV33 and HPV of another type of those determined by our PCR method in squamous cell carcinoma is approximately 55%, 2%, and less than 10%, respectively [4,13]. Thus, the probability to have 2 tumors with HPV of undetermined type in our series would be less than 1%.

### FGFR3 mRNA expression is higher in tumors with S249C FGFR3 mutation

**Figure 1:**
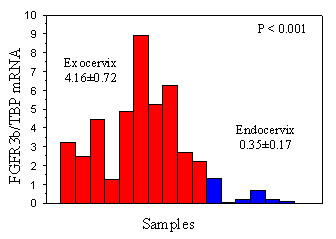
Schematic results of FGFR3b expression level in normal exocervix (n = 10) and normal endocervix (n = 7). FGFR3b expression is significantly higher in exocervix compared to endocervix.

**Figure 2:**
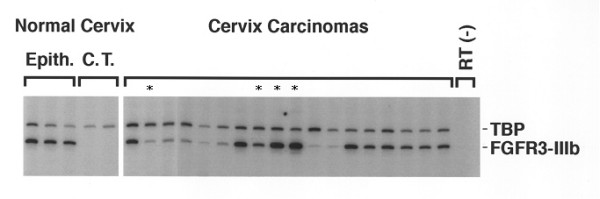
FGFR3b mRNA expression in normal cervix and invasive cervical carcinoma, using semi-quantitative RT-PCR with TBP as reference gene. The level of FGFR3b mRNA expression was measured in 3 normal exocervix epithelia, 2 underlying connective tissues (C.T.), 4 invasive cervical carcinomas with S249C FGFR3 mutation (*) and 14 invasive cervical carcinomas with wildtype FGFR3.

S249C FGFR3 mutation has been initially described in constitutional DNA from individuals with thanatophoric dysplasia [14]. It is suggested that activation of FGFR3 results from formation of intermolecular disulfide bonds between 2 mutant FGFR3 monomers. In order to get insight about association between S249C FGFR3 mutation and expression, we analyzed FGFR3b expression by semi-quantitative PCR in normal cervical samples and in invasive carcinomas (Figures 1, 2 and data not shown). In 17 normal cervix samples, FGFR3b/TBP mRNA ratios was 4.16 ± 0.72 in exocervical specimens and 0.35 ± 0.17 in endocervical specimens (Figure 1). Difference in of FGFR3b/TBP mRNA ratio means in exocervix and endocervix was statistically significant (P < 0.001, Mann-Whitney U test). FGFR3b mRNA levels was determined in 62 cervical carcinoma cases, including the 4 S249C mutated cases. Tumors with S249C mutation showed a significantly higher FGFR3b/TBP mRNA ratio than tumors without the S249C mutation (30.92 ± 21.74 *vs. *2.27 ± 0.36, *P *= 0.002, Mann-Whitney U test) (Figure 2).

**Figure 3:**
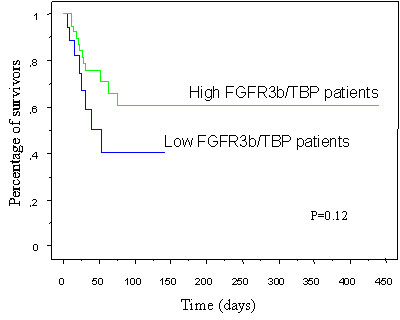
Survival stratified for the FGFR3b/TBP levels of invasive cervical carcinoma. Kaplan-Meier survival curves compare the cumulative probability of survival after diagnosis among patients with low FGFR3b/TBP levels to those with high FGFR3b/TBP levels.

We then sought for a correlation between survival and FGFR3b expression level. When patients were subdivided into 2 or 3 groups of equal size according to FGFR3b/TBP levels in cervical carcinoma, there was no statistically significant difference in survival between these groups. However, when patients were subdivided into 3 groups, there was a trend for patients with low levels of FGFR3b/TBP to have a shorter survival compared to the 2 other groups of patients with higher levels of FGFR3b/TBP (*P *= 0.12, Log-rank test) (Figure 3).

### Differentially expressed genes in FGFR3 mutated tumors compared to FGFR3 wildtype tumors

Finally, we used gene expression data from Affymetrix oligonucleotide microarray hybridization of invasive cervical carcinomas [15]. As all tumors with FGFR3 mutations were of the squamous type, we only used gene expression profiles from squamous cell carcinomas to compare tumors of same histological type and to minimize gene expression differences caused by different histology. We compared expression profiles of 3 FGFR3 mutated cervical carcinomas with the expression profiles of 17 wildtype FGFR3 cervical carcinomas, using SAM analysis as described in the methods section. With a threefold differential cutoff, we found 262 probe sets expressed at higher level and 552 probe sets at lower levels in FGFR3 mutated tumors compared to wildtype FGFR3 tumors (see additional files 1 and 2). To restrict the number of genes, we arbitrarily set a cutoff q value at 0.20 for higher expressed probe sets to end up with 61 probe sets, corresponding to 51 different known genes (Table 3). Genes with a role in regulation of transcription were the most frequently represented, including POU6F2, NHLH2, NR1D1, and ST18. FGFR3 was among the differentially expressed genes and was found upregulated, in agreement with RT-PCR results.

**Table 3:** Genes with higher expression in FGFR3 mutated cervical carcinomas compared to wildtype FGFR3 cervical carcinomas.

**Gene Symbol**	**Gene Title**
ABCB9	ATP-binding cassette, sub-family B (MDR/TAP), member 9
ACACA	acetyl-Coenzyme A carboxylase alpha
ANKRD6	ankyrin repeat domain 6
APIN	APin protein
AVP	arginine vasopressin
C8A	complement component 8, alpha polypeptide
CACNA2D3	calcium channel, voltage-dependent, alpha 2/delta 3 subunit
CACNB2	calcium channel, voltage-dependent, beta 2 subunit
CALML3	calmodulin-like 3
CAMK1G	calcium/calmodulin-dependent protein kinase IG
CAMK2B	calcium/calmodulin-dependent protein kinase II beta
CCL13	chemokine (C-C motif) ligand 13
CPEB1	cytoplasmic polyadenylation element binding protein 1
DBP	D site of albumin promoter (albumin D-box) binding protein
DDO	D-aspartate oxidase
ED1	ectodermal dysplasia 1, anhidrotic
FBXO26	F-box only protein 26
FGFR3	fibroblast growth factor receptor 3
FTHFD	formyltetrahydrofolate dehydrogenase
GNAO1	guanine nucleotide binding protein (G protein), alpha activating activity polypeptide O
GRIK4	glutamate receptor, ionotropic, kainate 4
GUCA2B	guanylate cyclase activator 2B
HIG2	hypoxia-inducible protein 2
KCNE1L	potassium voltage-gated channel, Isk-related family, member 1-like
LPHN3	latrophilin 3
MDM2	Mdm2, transformed 3T3 cell double minute 2
MEP1A	meprin A, alpha (PABA peptide hydrolase)
MYF6	myogenic factor 6 (herculin)
NEFH	neurofilament, heavy polypeptide 200kDa
NHLH2	nescient helix loop helix 2
NR1D1	nuclear receptor subfamily 1, group D, member 1
NRN1	neuritin 1
OGDHL	oxoglutarate dehydrogenase-like
POU6F2	POU domain, class 6, transcription factor 2
PPFIA4	protein tyrosine phosphatase, receptor type, f polypeptide
PVRL1	poliovirus receptor-related 1
RAPGEF3	Rap guanine nucleotide exchange factor (GEF) 3
RHAG	Rhesus blood group-associated glycoprotein
RNF6	ring finger protein (C3H2C3 type) 6
SLC4A3	solute carrier family 4, anion exchanger, member 3
SLC6A15	solute carrier family 6 (neurotransmitter transporter), member 15
SLC6A8	solute carrier family 6 (neurotransmitter transporter, creatine), member 8
SLC7A8	solute carrier family 7 (cationic amino acid transporter, y+ system), member 8
SMARCA4	SWI/SNF related, matrix associated, actin dependent regulator of chromatin, subfamily a, member 4
ST18	suppression of tumorigenicity 18 (breast carcinoma) (zinc finger protein)
TFAP2B	transcription factor AP-2 beta
TRIM2	tripartite motif-containing 2
UPB1	ureidopropionase, beta
ZNF257	zinc finger protein 257
ZNF287	zinc finger protein 287
ZXDA	zinc finger, X-linked, duplicated A

## Discussion

Since its first description, the role of FGFR3 mutation in cervical carcinoma has been debated when some studies reported an absence of mutation in a total of nearly 200 cases analyzed [9,11]. Here we showed that FGFR3 is mutated in 5% (4 of 75 cases) of invasive cervical carcinomas from a French cohort of patients (including 3 cases previously reported [5]). When all study results are grouped, the overall FGFR3 mutation rate in cervical carcinoma worldwide is 1.7%.

In bladder neoplasias, FGFR3 mutation characterizes low-grade low-stage pTa bladder tumors and is rarely found in advanced stage bladder carcinoma [16,17]. The low rate of FGFR3 mutation in invasive cervical carcinoma could result from the same distribution pattern, FGFR3 mutation being found at higher frequency in intraepithelial non invasive lesions. Here, we show that no mutation was identified in 80 cervical squamous intraepithelial lesions.

To better characterize FGFR3 mutated cervical tumors and to find explanations for the apparent discrepancy in mutation rates, we looked for clinical and pathological features that may be specifically associated with FGFR3 mutation. We showed that FGFR3 mutated tumors are more likely to be associated with a non-HPV16/18 type and that patients with mutated FGFR3 cervical carcinoma are older than patients with wildtype FGFR3 tumor. Epidemiologic studies have found an increase in prevalence with age for minor HPV types, such as types 39, 52, and 58 [4]. Age and association with minor HPV types may thus not be independent characteristics of patients with FGFR3 mutated cervical tumor. We can hypothesize that variations in reported FGFR3 mutation rate are, at least in part, related to differences in HPV types and in age distribution of studied patients. We cannot exclude that variations in FGFR3 mutation rate could also be related to other parameters, such as ethnic differences.

To get more insight into the biological significance of difference in FGFR3 mutated tumors, we sought for gene expression of FGFR3 in cervical tissues. FGFR3b expression is significantly higher in normal exocervix compared to normal endocervix and in FGFR3 mutated tumors compared to FGFR3 wildtype tumors. It has been suggested that the substitution of serine to cysteine in codon 249 may result in formation of intermolecular disulfide bond in the extracellular domain of two mutants FGFR3, causing a constitutive activation of the receptor [14]. It has been reported that activated FGFR3 results in activation of Jak/STAT pathway with STAT1 and STAT5 phsphorylation [18,19]. Here, we show for the first time that activation of FGFR3 is associated with an increase in FGFR3b expression in S249C mutated tumors. In bladder carcinoma, we also found a significantly higher FGFR3b expression in mutated tumors compared to wildtype tumors (unpublished data). Supervised analysis of Affymetrix expression data using SAM algorithm identified a significant number of genes that are up- or down-regulated in FGFR3 mutated tumors. These genes are potential targets of activated FGFR3. The small number of tumors in one group (3 vs. 17) may account for a high 0.20 false discovery rate for genes at higher expression in FGFR3 mutated tumors. However, the presence of FGFR3 in this list provides some evidence for the significance of these genes.

Finally, we found that patients with low FGFR3b expression in tumors are more likely to have a shorter survival than patients with high FGFR3b levels. Dai et al reported similar findings in an immunohistochemical study of 73 cervical carcinomas [11]. They found that patients with intense FGFR3 immunolabeling in tumor cells had a better prognosis. These results, not statistically significant in both studies, require further investigations.

## Conclusion

In summary, we demonstrated that S249C FGFR3 mutation defines a small subset of invasive cervical carcinoma and is not found in precursor intraepithelial lesions. Albeit small in number, patients with FGFR3 mutation appear to have distinctive clinical and biological characteristics that may help in defining a population for FGFR3 mutation screening. Gene expression analysis suggests that FGFR3 mutated tumors have a different biology with higher FGFR3b expression and differentially expressed genes which are potential targets of activated FGFR3.

## Methods

### Tissue specimens

Seventeen normal cervical mucosa and 75 invasive cervical carcinoma samples were obtained from the Institut Curie Hospital. The normal cervical mucosa samples were collected by scraping the cervix surface with a razor blade from 13 surgical resection specimens (hysterectomy or conization). Localization of normal samples was the exocervix (n = 10) and the endocervix (n = 7). Cervical carcinoma samples were collected from 75 patients with a median age of 48 years (range 23–82 years). Twelve of these samples had been previously studied for FGFR3 mutation [5]. Clinical stages according to the International Federation of Gynecology Obstetrics (FIGO) staging system were IB for 38 patients, IIA for 6 patients, IIB for 21 patients, IIIA for 3 patients, and IIIB for 6 patients. No staging information was available for 1 patient. Median follow-up was 32 months. In 73 cases, carcinoma samples originated from the initial cervical biopsy before any treatment, except in one case in which the patient previously received radiotherapy treatment. In 2 cases, the tumor sample was a pelvic recurrence after treatment. Final histological diagnoses were invasive squamous cell carcinoma (n = 66) and invasive adenocarcinoma (n = 9). HPV status was determined as previously described [20]. Briefly, Southern blot hybridization was the first-step procedure, using specific probes for HPV 6, 11, 16, 18, 31, 33, 35, 39, 42, 45, 52 and 58. A PCR with consensus primers in the L1 open reading frame was performed on cases negative by Southern blot hybridization. HPV DNA sequences were detected in 66/75 specimens (88%): 43 HPV16, 12 HPV18, 2 HPV33, 1 HPV31, 1 HPV58, and 7 undetermined HPV types.

Eighty cervical intra-epithelial neoplasia (CIN) biopsy or conization samples have also been selected: 40 high-grade squamous intraepithelial lesions (HSIL) from the Institut Curie Hospital (20 cases associated with HPV and 20 cases not associated with HPV); 40 low-grade squamous intraepithelial lesions (LSIL) from Dr. Isabelle Cartier Pathology Laboratory (20 cases associated with HPV and 20 cases not associated with HPV). Median age was 28 years (range 18–62 years) for patients with LSIL and 36 years (range 19–59) for patients with HSIL.

All normal samples, invasive carcinomas and squamous intraepithelial lesions were immediately frozen in liquid nitrogen and stored at -80°C until used.

### SSCP analysis and direct DNA sequencing

PCR-SSCP analysis was carried out on exons 7, 10, 15, 20 of the FGFR3 gene because these exons harbor all the activating mutations found previously in bladder and cervix carcinomas and in most human skeletal disorders due to FGFR3 mutations [5,21,22]. Samples that showed mobility shifts in SSCP analysis were further analysed by direct bidirectional sequencing. SSCP and sequence analysis were performed as previously described [16].

### Semi-quantitative RT-PCR

Total RNA was extracted from each sample by caesium chloride ultracentrifugation. Messenger RNA levels were determined by multiplex semi-quantitative RT-PCR using TBP (TATA binding protein) as internal control. Complementary DNA synthesis, PCR and analysis were performed as described [23]. Twenty two cycles were performed for the coamplification of FGFR3b and TBP. Primer sequences for TBP (TFIID), and FGFR3b (XF2 and TMR1) were as described [23,24].

### Oligonucleotide microarray analysis

We used results from a global gene expression analysis of 30 primary invasive cervical carcinomas [15]. Histological type was squamous cell carcinoma (SCC) for 20 samples and adenocarcinoma (AC) for 10 samples. S249C FGFR3 mutation was associated with 3 of the analyzed SCCs. Complementary RNA target was prepared and labeled as described in the Affymetrix GeneChip Expression Analysis Technical Manual, and hybridized to U133A Affymetrix oligonucleotide array, representing 22,215 probe sets.

Gene expression data were normalized across all samples and all probe sets, and log_2_-transformed. Significance analysis of microarrays (SAM) [25] was used to perform the two-class comparison for differentially expressed genes between the 3 cervical SCCs with FGFR3 mutation and the 17 cervical SCCs with wildtype FGFR3. The K-nearest neighbor imputation was used to account for missing data within the dataset. Output criteria selected for SAM included at least threefold greater expression in the FGFR3 mutated samples as compared to the FGFR3 wildtype samples. The false discovery rate (q value) was arbitrarily set to 0.20.

### Statistical Analysis

All results are given as mean ± standard error of the mean unless otherwise specified. The Mann-Whitney U test was used to compare continuous variables. For survival analysis, patients who died because of the cervical cancer were categorized as deaths. All other clinical evolution were censored. Patients were subdivided into 2 or 3 groups of equal size, according to the level of FGFR3b/TBP expression in cervical carcinoma. Survival analysis was carried out using the Kaplan-Meier method. The difference between survival curves was analyzed by the log-rank test. Probability values of 0.05 or less were considered significant.

## Authors' contributions

CR found the relationship between the occurrence of FGFR3 mutations and an older age for the patients. He also analysed the microarray experiments. MHA performed the SSCP analysis. DC performed the RT-PCR analysis together with JB. JB prepared the normal and tumor cervical samples and performed the RT-PCR experiments together with DC. IC provided the cervical intra-epithelial neoplasia specimens. XSG set up the cervix tumor bank and initiated the work on cervical cancer. FR coordinated the work. CR, MHA and FR participated in the writing of the manuscript. All authors participated in the different discussions, the interpretation of the data, participated in the correction of the manuscript and approved the final version.

## Supplementary Material

Additional File 1 Raw data of gene expression analysis of 20 cervical carcinomas. Gene expression results of 20 cervical squamous cell carcinomas (SCC), using Affymetrix U133A array. Click here for file

Additional File 2 SAM analysis from gene expression data of 20 cervical carcinomas. genes differentially expressed in FGFR3 mutated tumors (n = 3) compared to FGFR3 wildtype tumors (n = 17), identified by SAM analysis. Click here for file
